# Adverse childhood experiences and substance misuse in young people in India: results from the multisite cVEDA cohort

**DOI:** 10.1186/s12889-021-11892-5

**Published:** 2021-10-23

**Authors:** G. S. Fernandes, A. Spiers, N. Vaidya, Y. Zhang, E. Sharma, B. Holla, J. Heron, M. Hickman, P. Murthy, A. Chakrabarti, D. Basu, B. N. Subodh, L. Singh, R. Singh, K. Kalyanram, K. Kartik, K. Kumaran, G. Krishnaveni, R. Kuriyan, S. Kurpad, G. J. Barker, R. D. Bharath, S. Desrivieres, M. Purushottam, D. P. Orfanos, M. B. Toledano, G. Schumann, V. Benegal

**Affiliations:** 1 Population Health Sciences, Bristol Medical School, University of Bristol, Bristol, BS8 2BN UK; 2 Department of Epidemiology and Biostatistics, Imperial College London, London, UK; 3 Centre for Addiction Medicine, National Institute of Mental Health and Neurosciences, Bangalore, India; 4 Centre for Population Neuroscience and Precision Medicine, Kings College London, London, UK; 5 Centre for Innovation in Mental Health, Department of Psychology, University of Southampton, Southampton, UK; 6 ICMR-Centre on Non-Communicable Diseases, Kolkata, India; 7 Department of Psychiatry, Post Graduate Institute of Medical Education and Research, Chandigarh, India; 8 Department of Psychiatry, Regional Institute of Medical Sciences (RIMS), Imphal, Manipur India; 9 Rishi Valley Rural Health Centre, Madanapalle, Chittoor, Andhra Pradesh India; 10 Epidemiology Research Unit, CSI Holdsworth Memorial Hospital, Mysore, India; 11 Department of Psychiatry and Medical Ethics, St John’s Medical College & Hospital, Bangalore, India; 12 Department of Psychiatry & Department of Medical Ethics, St. John’s Medical College & Hospital, Bangalore, India; 13 Department of Neuroimaging, King’s College London, London, UK; 14 NeuroSpin, CEA, Université Paris-Saclay, Paris, France

**Keywords:** Adverse childhood experiences, physical abuse, sexual abuse, Indian children, substance misuse, cVEDA

## Abstract

**Background:**

Adverse childhood experiences (ACEs) increases vulnerability to externalising disorders such as substance misuse. The study aims to determine the prevalence of ACEs and its association with substance misuse.

**Methods:**

Data from the Consortium on Vulnerability to Externalising Disorders and Addictions (cVEDA) in India was used (*n* = 9010). ACEs were evaluated using the World Health Organisation (WHO) Adverse Childhood Experiences International Questionnaire whilst substance misuse was assessed using the WHO Alcohol, Smoking and Substance Involvement Screening Test. A random-effects, two-stage individual patient data meta-analysis explained the associations between ACEs and substance misuse with adjustments for confounders such as sex and family structure.

**Results:**

1 in 2 participants reported child maltreatment ACEs and family level ACEs. Except for sexual abuse, males report more of every individual childhood adversity and are more likely to report misusing substances compared with females (87.3% vs. 12.7%). In adolescents, family level ACEs (adj OR 4.2, 95% CI 1.5–11.7) and collective level ACEs (adj OR 6.6, 95% CI 1.4–31.1) show associations with substance misuse whilst in young adults, child level ACEs such as maltreatment show similar strong associations (adj OR 2.0, 95% CI 1.1–3.5).

**Conclusion:**

ACEs such as abuse and domestic violence are strongly associated with substance misuse, most commonly tobacco, in adolescent and young adult males in India. The results suggest enhancing current ACE resilience programmes and ‘trauma-informed’ approaches to tackling longer-term impact of ACEs in India.

**Funding:**

Newton Bhabha Grant jointly funded by the Medical Research Council, UK (MR/N000390/1) and the Indian Council of Medical Research (ICMR/MRC-UK/3/M/2015-NCD-I).

**Supplementary Information:**

The online version contains supplementary material available at 10.1186/s12889-021-11892-5.

## Introduction

The World Health Organisation (WHO) reports that early childhood adversity such as child maltreatment is widespread, with 1 in 4 adults reporting being physically abused as children globally [1]. Physiological and neurobiological studies have found that such childhood adversities can lead to changes of neural networks, impaired nervous, endocrine and immune systems development resulting in chronic physiological dysfunction and damage [2]. Adverse childhood experiences (ACEs) include direct (physical abuse, sexual abuse, emotional abuse, physical neglect and emotional neglect), indirect (bullying, parental violence, parental substance misuse, parental incarceration, parental divorce/separation and parental suicide or self-harm) and extended harms (collective violence, community violence) to children [3, 4]. These adversities can be assessed either retrospectively or prospectively, using a range of validated tools such as the World Health Organisation’s Adverse Childhood Experiences International Questionnaire (ACEIQ) or clinician-based reports [5]. In adults who retrospectively report being abused or neglected as children, there is evidence of an association with engaging in multiple risk behaviours such as harmfully using tobacco, drugs and alcohol [3, 6]. Experiencing such adversities in childhood that are prospectively recorded, can also impact on externalising behaviours such as substance misuse in adolescence or young adulthood itself [6], although there is limited evidence of these immediate effects, particularly in lower- and middle-income countries [7].

Over the past 40 years, research has identified a myriad of factors associated with an increased risk of abuse and neglect. These include parent’s personalities, lifestyles and social contexts and family structures and dynamics [8, 9]. The complex and multifaceted nature of child maltreatment and other adversities can be encompassed by several socioecological frameworks, in which adversities are determined by factors at work in the individual, in the family, in the community and culture, and that these determinants are nested within one another. Whilst this granularity may not always be documented, in some countries, like India, any detailed, national data on ACEs is lacking [10]. This is poignant too, given the main criticism of the traditional ACEs models is overlooking the socioeconomic contexts, such as child poverty and social or gender-based inequalities, within which ACEs exist, re-occur and cluster [11]. There exist only a handful of studies on the prevalence of ACEs in specific communities [7]. Damodaran and Varghese [10] compiled data on ACEs in youth based in Kerala (South India) and stated that in a sample of 600 young people, 91% reported at least one ACE and over half the population reported three or more ACEs. Most ACEs within an Indian context have received little attention except for child sexual abuse (CSA) [7]. It is estimated that every second child is exposed to sexual abuse and violence [12]. These figures are considered underestimates because of limited surveillance of direct ACEs such as physical abuse as well as CSA, due to stigma particularly affecting girls; underreporting of cases by healthcare and police authorities; and, varying prevalence rates by geography (e.g. urban versus rural settings) and communities (school-based, trafficking victims etc) [13].

The impact of ACEs is not limited to chldhood and can affect risk behaviours and opportunities such as education or employment potentials, well into adulthood [3]. There exists a strong body of evidence supporting the dose-reposponse relationship between ACEs and development of a substance use disorder or harmfully using substances [14]. However, not all ACEs are predictors of developing substance misuse behaviours and certainly, vulnerability to developing alcohol and other substance use disorders are not uniformly distributed across the population and may be affected by the occurrence of a single ACE, multiple ACEs or re-occurrence of ACEs and appear to be associated across different races/ethnicities. A study of college students in America found that while ACEs were associated with substance misuse, there was significant ethnic variation in ACE exposures between non-Hispanic White, Hispanic and African/American Black students. The authors also report a dose-response relationship between ACEs and specific substances, namely tobacco and alcohol [15]. However, applying this lens to India is limited as there are unique geographical, cultural and socioeconomic factors at play, given that most drug users seeking treatment were introduced to drugs at 15 years or younger [10, 16]. In India, externalising disorders, such as alcohol abuse and alcohol dependence, contribute significantly to the global burden of disease [10]. Alcohol attributable mortality in India is almost twice the rate of higher income countries, with lifetime alcohol use in school-attending adolescents associated with tobacco and drug use, and a history of sexual abuse [16]. One in four of Indian adolescents (aged 13–15 years) also reports having used or currently using tobacco in different forms (e.g. chewing and smoking)(12) and cannabis is the most commonly used prohibited substance among school students, street children and working adolescents in India [17]. Squeglia and colleagues [18] report that there exist several neuropsychological and neuroimaging studies that have highlighted neural vulnerabilities that lead to the initiation of substance misuse in adolescence. Underage alcohol and drug use is a leading public health and social problem for individual adolescents, their families and communities, and a country. With the world’s largest population of adolescents, at over 200 million, India has a huge ‘at risk’ demographic that is vulnerable to substance misuse and addiction [19, 20]. To date, we have found no studies evaluating the prevalence of ACEs at a national level and the impacts of ACEs on substance misuse in Indian adolescents and young adults. Mapping ACE prevalence and substance misuse in India would help inform prevention and management strategies lead to evidence-informed trauma practices.

We propose to evaluate these research questions by using data from the Consortium on Vulnerability to Externalising Disorders and Addictions study (cVEDA), the largest paediatric and young adult study of factors affecting neurodevelopment on the Indian subcontinent [15, 21]. The study aims to: determine the prevalence of ACEs in India by age-band, sex and recruitment site, and determine the association between ACEs and hazardous and harmful substance misuse, specifically tobacco, alcohol and cannabis.

## Methods

### Setting

The Consortium on Vulnerability to Externalising Disorders and Addictions (cVEDA) consists of seven study recruitment centres and corresponding catchment areas including: Imphal (Manipur); Asansol (West Bengal); Mysore (Karnataka); National Institute of Mental Health and Neurosciences [NIMHANS] Bangalore (Karnataka); Post Graduate Institute of Medical Education and Research [PGIMER] (Chandigarh, Punjab and Haryana); Rishi Valley (Madanapelle, Andhra Pradesh); Saint John’s Research institute [SJRI], Bangalore (Karnataka).

The cVEDA study began in February 2016 and 9 months were spent in the study set up including staff recruitment, translations of study instruments into 7 Indian regional languages, setting up digital data capture platforms, training of recruitment and assessment teams, and quality control measures. Recruitment of participants then officially began in October 2016. The target sample sizes at each recruitment centre were decided based on the capacity to recruit individuals over a 3-year recruitment period, given the site lead’s understanding about ground realities and experience from past studies. The same represents five geographically, ethnically, and socio-culturally distinct regions including a variety of environmental risks: toxic exposures (coalmines), slum-dwellers, socio-political conflict zones and urban and rural areas.

Full details on recruitment catchment areas, informed consent and inclusion and exclusion criteria have been previously published in protocol papers [15, 21]).

### Ethics

The study protocol was approved by the ethics committees of the National Institute of Mental Health and Neurosciences (NIMHANS) in Bangalore, India (REF Item no. V99, SI. 7.08, Behavioural Sciences) and all regional collaborating sites. The Indo-UK collaboration was approved by the Health Ministry Screening Committee of the Ministry of Health and Family Welfare, Government of India. The study was conducted in accordance with the Declaration of Helsinki (1964 and later versions).

All assessments involved dimension and categorical phenotypic characterization. The questionnaires and assessment protocols were translated (and back translated using standard WHO protocols) from English into seven Indian languages (Hindi, Kannada, Telugu, Tamil, Manipuri, Bengali and Punjabi) for use across the seven recruitment centres. Age-appropriate instruments were used to capture socio-demographic information, temperament, environmental exposures, parenting, psychiatric morbidity, and neuropsychological functioning.

### Adverse Childhood Experiences Assessment

ACEs were assessed in cVEDA using the ACE-International Questionnaire (ACEIQ), a World Health Organisation (WHO) 43-item questionnaire that measures all aspects of direct and indirect ACEs [22].

The domains of the ACE-IQ include: *relationship with parents or guardians* (e.g. having enough food to eat, parents being too drunk or intoxicated to provide adequate care), *family environment* (substance misuse, divorce, incarceration or death of family member, abuse and neglect), *peer violence* (experience bullying or being involved in a physical fight), *witnessing community violence* (witnessing someone being stabbed, shot or threatened in the community) and *exposure to war/collective violence* (e.g. moving because of war, conflict, political conflicts). Given the myriad socioecological frameworks that exist in the literature, several authors had advocated examining factors in each of these four domains: the children (individual level), the family background (ontogenic or microsystem level), the community (exosystem level) and the wider society (macrosystem level) [23]. We adopted this approach in our analysis plan and categorised categorised ACE-IQ into four main ACE components/levels based on the socioecological model of child maltreatment [9], defined as follows:

- Child Maltreatment (individual level) comprised of physical abuse, emotional abuse, sexual abuse, emotional neglect or physical neglect.
- Family (microsystem level) comprised of domestic violence, parental substance misuse, parental incarceration, parental divorce/separation, parental with mental health conditions
- Community (exosystem level) comprised of experiences of bullying or community violence
- Collective (macrosystem level) comprised of experiences of collective violence

To calculate a binary ACE score for child maltreatment, we used the 4 questions on neglect (physical and emotional), and 8 questions on abuse (physical, sexual and emotional). The binary ACE score for family level ACEs was collated using the 9 questions on family dysfunction, peer violence and domestic violence. The binary score for community violence ACEs consisted of 3 questions on being witness to stabbings, beatings, shootings and being threatened with a knife or gun. A binary score for collective violence ACEs was collated using the 4 questions on experiences of witnessing homes and families being displaced, beaten up or killed by military, police or militia groups. Participants had the option of answering ‘yes’, ‘no’, or ‘refuse’ to some questions and ‘many times’, ‘a few times’ and ‘never’ to other questions. For each individual, the response to each question represents an exposure to an ACE and was counted as one event when the participant responses ‘yes’, ‘many times’ or ‘a few times’ on a question. All responses pertain to the entire period of childhood or ‘ever’ time frame for children and adolescents (6–18 years), and to the first 18 years of life for young adults (18–23 years) in cVEDA.

### Substance Misuse

The use of substances was assessed using the Alcohol, Smoking and Substance Involvement Screening Test (ASSIST) questionnaire, developed by the WHO and screens for all levels of hazardous and harmful substance misuse [24]. The ASSIST is an 8-item questionnaire spanning the past 3 months and lifetime; and screens for the use of the following substances: tobacco products, alcohol, cannabis, cocaine, amphetamine-type stimulants, sedatives and sleeping pills, hallucinogens, inhalants, opioids and other drugs. The ASSIST determines a risk score for each substance which falls into a low-risk, moderate- risk (hazardous use) or high-risk category (harmful use//indicating substance dependence) which determines the appropriate level of intervention required. We created and defined a category for the most commonly used substances [19, 20] namely tobacco, alcohol and cannabis (TAC) by combining hazardous and harmful use of these substances into a dichotomous measure.

### Confounders

We included age, sex and socioeconomic status (SES) as potential confounders. SES was determined on the basis of housing quality – a combination of roof type, wall type and floor type (grass/straw/leaves/reeds/bamboo, mud/unburnt brick, canvas/cloth, other untreated/weak material, tiles/slate, burnt brick/stone, iron/zinc/metal sheet and, cement). We dichotomised SES on the basis of housing composition with lower-quality materials (grass/reeds, mud and canvas/cloth) indicating lower SES whilst tiles/slate metal and cement indicated higher SES as used in the National Sample Survey of India [25]. We also enquired after family structure within the household defined as nuclear family (parents and children), joint family (extended family) or other (three generations of the same family within the household). We dichotomised family structure by combining joint family with extended family settings versus nuclear family households. Urbanisation was determined by the recruiters based on geographical location and given a value of urban, rural, slum or missing (not disclosed). This was dichotomised by combining rural and slum households versus urban households. Home ownership (owned/rented) was also recorded as a dichotomous variable when individual participants were asked about personal anthropometrics and socioecological details at the recruitment visit.

### Statistical Analysis

Descriptive data on the prevalence of ACEs by sex, recruitment site, and age-band (frequencies, % and chi^2^ tests) were presented. Given the differences in prevalence of ACEs and substance access, legislation and use by different age groups (e.g. children, adolescents and young adults), we analysed these data by the age bands identified in the study protocol:: adolescents or C2 (aged 13–17 years) and finally, young adults or C3 (aged 18–23 years). However, cVEDA effectively contains three different age-cohorts (C2 and C3 detailed above) as well as C1 (aged 6–12 years). However, the scarcity of substance use precluded any analysis in C1. The term ‘adolescents’ from now onwards will refer to the cVEDA age band C2, ages 13–17 years, while the term ‘young adults’ will refer to the cVEDA age band C3, ages 18–23 years.

Whilst all cVEDA recruitment sites share a single study design as well as the same data collection period there are likely to be many sociocultural and political differences between sites, including many differences for which we do not possess site level data. In the light of this we decided to employ meta-analytic techniques (further details below) to derive pooled estimates of the association between ACEs and substance misuse rather than working with a single combined sample. We calculated both the unadjusted and adjusted odds ratio (OR) and associated 95% confidence intervals (CI) for the association between the four ACE groups and hazardous and harmful substance misuse. We used Stata’s firthlogit command rather than traditional logistic regression to guard against the problem of perfect prediction and to allow the same set of confounders to be included for each site despite differences in the confounder distribution and/or outcome prevalence. Firth logistic regression fits logistic models by penalised maximum likelihood regression (PMLE) and has been recommended as a solution to small sample sizes [26] where such problems are more likely to occur.

### Further details on meta-analytic methods employed

A random effects meta-analysis was employed to account for perceived sites differences. Meta-analysis is more commonly used when analysing results from clinical trials however it can also be used to pool the results of observational studies [27–29]. This technique allowed us to estimate the association between ACEs and TAC within each site and understand these in the context of other sites/regions i.e., whether effect sizes are consistent across cVEDA data. In a random-effects meta-analysis, we assume not one true effect i.e., the fixed effect, but instead distribution of effects between ACEs and TAC. Given the diversity of India’s population, the one true effect assumption may not be accurate, and our approach allows us to accommodate and measure the heterogeneity.

Given that participant data was available and we did not need to rely on summary information from each site, we used Stata’s ipdmetan [30] routine which permits the use of individual patient data (IPD). Due to the small number of study sites in cVEDA as well as and the anticipated heterogeneity between sites, we used a Hartung-Knapp-Sidik-Johnkman (HKSJ) modification, which uses an estimate of the between-site variance to yield more robust estimates of variance [31]. Simulation studies have shown that standard Dersimonian-Laird random effects meta-analysis are more likely to given anti-conservative (narrow) confidence intervals [32, 33].

We also present the estimate of variance of the true effect size distribution (between-site variance) using T^2^ or tau^2^ and Cochrane’s Q heterogeneity statistics, df the degrees of freedom and associated *p* value. As the differences between studies was more than a sampling error, this approach accounts for both within-site and between-site differences to produce a summary effect as the mean effect and improving its generalisability.

We used complete-cases analysis approach for our analysis as we had few missing data for individual participants (Table 1). All analysis was undertaken using STATA 16.

**Table 1:** Sociodemographic characteristics of the cVEDA adolescent and young adult responders with indicators if included as a confounder per ACE level (*)

Variable	N (%)	Confounder per ACE level			
		Child	Family	Community	Collective
**Age band**					
*Adolescents*	3369	–	–	–	–
*Young adults*	2751				
**Sex**		*			
*Males*	2938 (48.01)				
*Females*	3182 (51.99)				
**Geometrics**		*	*	*	*
*Urban*	3075 (50.24)				
*Rural*	2684 (43.87)				
*Missing*	361 (6.8)				
**Family Structure**		*	*		
*Nuclear*	4656 (76.07)				
*Joint Family*	1101 (17.99)				
*Missing*	363 (5.9)				
**Housing Quality**^a^		*	*	*	*
*Low-quality (thatch, mud)*	642 (10.49)				
*High-quality (concrete, tiles, brick, cement, etc)*	5034 (82.25)				
*Missing*	444 (7.25)				
**Home Ownership**		*			*
*Own*	4497 (73.48)				
*Rented*	1623 (26.52)				

^a^Housing quality consists of structural materials used to construct the house floors, walls and roof

## Results

6120 adolescent and young adult participants completed baseline assessments. 2890 children also completed the baseline assessment but reported only few cases of substance misuse (*n* = 2, 0.07%) and hence were excluded from secondary analyses (Additional file 1: Appendix 1).

Table 1 shows sociodemographic data of all participants. 52.2% of the cohort were female and the majority of participants lived in urban areas (47.3%) and rural areas (37.9%). Only 8% of the cohort reported living in a slum area whilst 6.8% did not disclose such geometric data. The majority of adolescent and young adult participants lived in a nuclear family setting (77.5%) and reported high housing-quality (81.1%).

### Adverse Childhood Experiences

Table 2 shows that all types of child level ACEs range from 8.9–39.8% for all types of neglect and abuse except for sexual abuse. The overall reported prevalence of sexual abuse is 1.6% and reported more by girls than boys (1.9% vs. 1.6%, *p* < 0.001) particularly in the young adult age band (C3). In terms of family level ACEs- parental divorce rates differed by age-band (*p* < 0.001) with 9.1% of the young adult group reporting parental divorce or separation compared to 5.6% of children, but there was no effect of sex (*p* = 0.24). Overall, domestic violence was witnessed by 33% of the sample and this was equivalent by sex (33.8% in males, 32.4% in females, *p* = 0.25). There was also a marked effect by sex with more males reporting community violence (26.7% vs. 24.0%) and collective violence (4.9% vs. 2.7%) compared to females (*p* = 0.008 and *p* < 0.001 respectively). Higher rates of substance abuse were reported in Bangalore (NIMHANS) (50.5%) and Chandigarh (PGIMER) (59.8%) as the participants from these sites were partly recruited from children of treatment seekers at de-addiction services. Overall, Imphal has the highest rates of child level ACEs (94.9%), family level ACEs (85.7%), community level ACEs (82.1%) and collective level ACEs (20.2%) (Additional file 2: Appendix 2).

**Table 2:** The prevalence of ACEs by age band and sex in the cVEDA cohort

Type of ACE	Total sample n (%)	Age Band n (%)			***P*** value	Sex n (%)		***P*** value
		C1	C2	C3		Male	Female	
Physical Abuse	2878 (31.9%)	862 (29.8%)	1228 (36.4%)	782 (28.4%)	*p* < 0.001	1443 (33.5%)	1429 (30.4%)	*p* < 0.001
Sexual Abuse	161 (1.8%)	26 (0.9%)	50 (1.5%)	85 (3.1%)	*p* < 0.001	69 (1.6%)	92 (1.9%)	*p* < 0.001
Emotional Abuse	3589 (39.8%)	1003 (34.7%)	1490 (44.2%)	1096 (39.8%)	*p* < 0.001	1730 (40.2%)	1859 (39.5%)	*p* < 0.001
Emotional Neglect	1028 (11.4%)	254 (8.8%)	431 (12.8%)	343 (12.5%)	*p* < 0.001	562 (13%)	466 (9.9%)	*p* < 0.001
Physical Neglect	803 (8.9%)	200 (6.9%)	325 (9.6%)	278 (10.1%)	*p* = 0.001	431 (10%)	372 (7.9%)	*p* = 0.001
**Child Level**^a^	4339 (48.2%)	1250 (43.3%)	1778 (52.8%)	1311 (47.7%)	*p* = 0.001	2093 (48.6%)	2246 (47.7%)	*p = 0.38*
Parental Divorce	703 (7.8%)	161 (5.6%)	293 (8.7%)	249 (9.1%)	*p* < 0.001	355 (8.3%)	348 (7.4%)	*p* = 0.241
Parental Mental Illness	402 (4.5%)	99 (3.4%)	154 (4.6%)	149 (5.4%)	*p* = 0.021	207 (4.8%)	195 (4.1%)	*p* = 0.007
Parental Substance Abuse	2350 (26%)	776 (26.9%)	885 (26.3%)	689 (25%)	*p* < 0.001	1311 (30.5%)	1039 (22%)	*p* < 0.001
Parental Incarceration	142 (1.6%)	42 (1.5%)	58 (1.7%)	42 (1.5%)	*p* < 0.001	72 (1.7%)	70 (1.5%)	*p* = 0.01
Domestic Violence	2975 (33%)	789 (27.3%)	1252 (37.2%)	934 (34%)	*p* < 0.001	1452 (33.8%)	1523 (32.4%)	*p* = 0.25
**Family Level**^a^	4238 (47%)	1259 (43.6%)	1682 (49.9%)	1297 (47.2%)	*P = 0.006*	2140 (49.7%)	2098 (44.6%)	*p* < 0.001
Bullying	763 (8.5%)	184 (6.4%)	318 (9.4%)	261 (9.5%)	*p* = 0.079	475 (11%)	288 (6.1%)	*p* < 0.001
Community Violence	2278 (25.3%)	511 (17.7%)	941 (27.9%)	826 (30%)	*p* < 0.001	1151 (26.7%)	1127 (24%)	*p* = 0.008
**Community Level**^a^	2617 (29.1%)	622 (21.5%)	1080 (32.1%)	915 (33.3%)	*p* < 0.001	1349 (31.4%)	1268 (27%)	*p* < 0.001
Collective Violence	338 (3.8%)	34 (1.2%)	143 (4.2%)	161 (5.9%)	*p* < 0.001	210 (4.9%)	128 (2.7%)	*p* < 0.001

^a^Child level is an amalgamation of physical abuse, sexual abuse, emotional abuse, emotional neglect and physical neglect; family level is an amalgamation of parental divorce, parental mental illness, parental substance abuse, parental incarceration and domestic violence; community level is comprises of bullying and community violence

### Meta-analysis

**Fig. 1:**
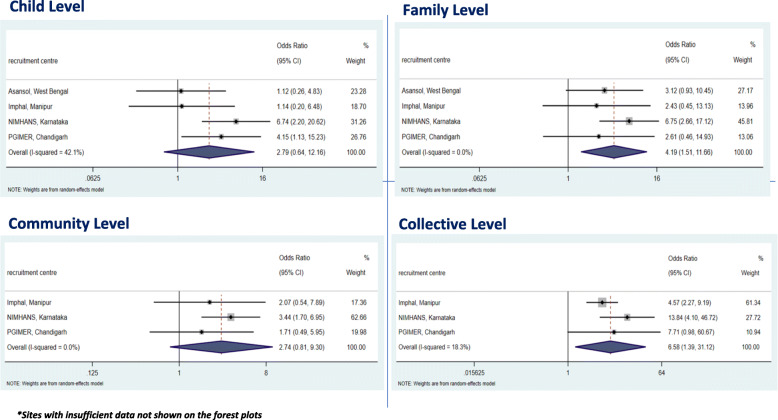
Forest plot outputs for TAC outcomes from adjusted IPD meta-analysis in the adolescent group. *Sites with insufficient data not shown on the forest plots

**Fig. 2:**
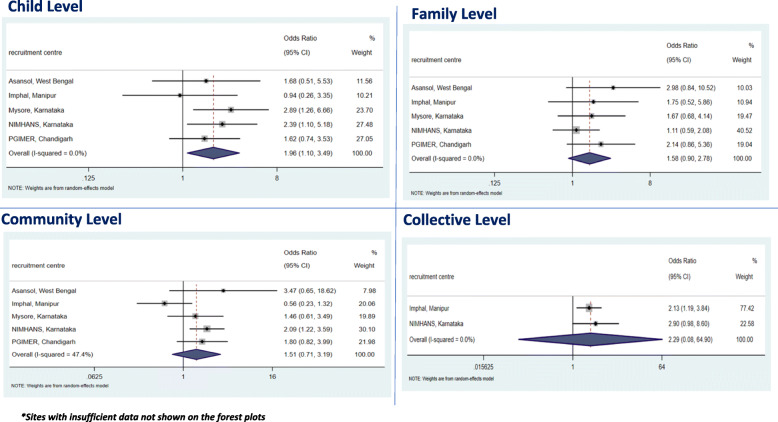
Forest plot outputs for TAC outcomes from adjusted IPD meta-analysis in the young adult group. *Sites with insufficient data not shown on the forest plots

The overall meta-analysis results and individual cVEDA site findings with corresponding forest plots for all substance misuse (TAC) are presented in Fig. 1 and Fig. 2 respectively. These unadjusted and adjusted results with heterogeneity estimates are presented in Tables 3 for the adolescents and young adults, respectively. Sites with insufficient data were not presented in the forest plots as there were limited or no TAC outcomes (Additional file 3: Appendix 3). All individual substance misuse for adolescents and young adults are included in Additional file 4: Appendix 4.

**Table 3:** Unadjusted and adjusted effect sizes and heterogeneity measures for ACE levels and TAC outcomes in adolescents and young adults

ACE level	Unadjusted Overall Effect (OR)	Unadjusted 95% CI	Adjusted Overall Effect (OR)	Adjusted 95% CI	Cochrane’s Q			I^2^ (%)	tau^2^
					*Q*	*df*	*P value*		
*Adolescents* TAC^*^									
Child level	4.06	2.46–6.70	2.79	0.64–12.16	5.18	3	0.16	42.1	0.36
Family level	4.95	2.87–8.56	4.19	1.50–11.66	1.92	3	0.59	0	0
Community level	3.36	2.15–5.25	2.74	0.80–9.30	1.13	2	0.57	0	0
Collective level	7.28	4.41–12.00	6.58	1.39–31.12	2.45	2	0.29	18.3	0.09
*Young Adults*									
Child level	4.43	3.22–6.09	1.96	1.10–3.49	2.67	4	0.62	0	0.00
Family level	4.05	2.97–5.52	1.58	0.89–2.78	2.67	4	0.62	0	0.00
Community level	2.77	2.12–3.62	1.51	0.71–3.19	7.60	4	0.10	47.4	0.16
Collective level	3.17	2.13–4.74	2.29	0.08–64.90	0.24	1	0.63	0	0

^*^TAC is a binary outcome indicating presence of any one or more of substances misused: tobacco, alcohol and cannabis

Alcohol misuse frequency is 26 in this age band

### Adolescents

Those adolescents who experienced child level ACEs (OR 4.06, 95% CI 2.46–6.70), family level ACEs (OR 4.95, 95% CI 2.87–8.56), community level ACEs (OR 3.36, 95% CI 2.15–5.25) and collective level ACEs (OR 7.28, 95% CI 4.41–12.00) were found to have an overall higher risk of hazardous and harmful use of substances (TAC) in cVEDA (Table 3). However, when adjusting for covariates, these results were attenuated for child level ACEs (adjusted OR 2.79, 95% CI 0.64–12.16) and community level ACEs (adjusted OR 2.74, 95% CI 0.80–9.30) but not for family level ACEs (adjusted OR 4.19, 95% CI 1.50–11.66) or collective level ACEs (adjusted OR 6.58, 95% CI 1.39–31.12) (Table 3).

Figure 1 shows the results of adjusted IPD meta-analysis for individual cVEDA sites with corresponding heterogeneity scores showing that despite the variation in recruitment centres, there was little to no heterogeneity for each level of ACE with I^2^ ranging from 0 to 42.1%.

At specific sites such as NIMHANS (Fig. 1), after adjustment, an experience of child level ACEs such as abuse meant that an adolescent was almost 7 times more likely to misuse substances (OR 6.74, 95% CI 2.20–20.62); an experience of family level ACEs such as parental substance misuse meant that they were almost 7 times more like to misuse substances (OR 6.75, 95% CI 2.66–17.12); and an experience of community level ACEs such as witnessing community violence meant that they were 3 times more likely to misuse substances (OR 3.44, 95% CI 1.70–6.96).

### Young Adults

The young adults who experienced child level ACEs (OR 4.43, 95% CI 3.22–6.09); family level ACEs (OR 4.05, 95% CI 2.97–5.52); community level ACEs (OR 2.77, 95% CI 2.12–3.62); and collective level ACEs (OR 3.17, 95% CI 2.13–4.74) were found to have an overall higher risk of misusing substances in cVEDA (Table 3). After adjustment for confounders, the associations were attenuated for all ACE levels and there remained only an association between child level ACEs (OR 1.96, 95% CI 1.10–3.49) with TAC misuse (Table 3). In this age band, females reported more child level ACEs, family level ACEs and community level ACEs compared to males, but not collective level ACEs. However, in terms of the substance misuse outcomes, more males reported TAC (86.78%) than females (13.22%).

Figure 2 shows the results of the adjusted IPD meta-analysis for individual cVEDA sites with corresponding site weighting and heterogeneity scores.

The results (Fig. 2) show that there is homogeneity across all sites for all levels of ACEs and TAC outcomes in young adults except for community level ACEs. Similar to the adolescent group, we note the effect of collective level ACEs on TAC outcomes (OR 2.13, 95% CI 1.19–3.84) in Imphal compared with other cVEDA sites.

## Discussion

Almost 1 in 2 participants reported child maltreatment ACEs such as physical abuse and 1 in 2 reported family level ACEs such as domestic violence. 1 in 3 participants reported experiencing some form of community level ACEs whilst 4% of the sample reported experience collective violence in form of political and economic instability in their home communities. Except for sexual abuse, males report more of every individual childhood adversity and ACE level (child, family, community and collective) compared with females. Our findings also suggest that males are more likely to report using substances hazardously and harmfully compared with females. In cVEDA adolescents, after adjustment, family level ACEs and collective level ACEs show an association with substance misuse. However, in cVEDA young adults, only child level ACEs show an association with substance misuse, after adjusting for confounders, whilst this association was attenuated for other ACE levels.

### Adverse Childhood Experiences: Global and Indian perspectives

Despite almost half the cVEDA cohort reporting at least one form of child maltreatment ACE, this is lower when compared with the largest nationwide study on childhood adversity [34]. The study found that 68.9% of children reported physical abuse; 53% reported sexual abuse; 48.4% reported emotional abuse and 70.6% reporting neglect [34]. However, cVEDA prevalence figures for child maltreatment were higher compared to other Indian school-based studies [35, 36] where 25–40% of child experience physical abuse; 12.7–48% experience sexual abuse; 45–52% experience emotional abuse and 60% experience neglect. These discrepancies can be attributed to the wide variation in: sample sizes; methods of child maltreatment assessment; exposure time frame; and geometric differences between urban, rural and slum regions. Child maltreatment prevalence for cVEDA are much higher when compared with global data on physical abuse (23%), emotional abuse (36%) and physical neglect (16%) but not for sexual abuse (18% in girls, 8% in boys) [1].

Overall, our findings of sexual abuse are low (1.8%) compared with previous studies on sexual abuse in India which reported prevalences of 17–74% [7, 10, 12, 13]. However, our figures are comparable to a more recent study by Beattie and colleagues who reported a prevalence of 1.6% in school-based girls. These figures, as with cVEDA, are thought to be underreported as in Beattie’s study [37], where almost 5% of girls refused to answer questions on sexual abuse and a further 8% revealed they were hesitant to report sexual harassment. This supports previously published ACE literature in India where sexual abuse is more prevalent among girls due to sex disadvantages, discrimination and inequality [7].

### Other Evidence

In cVEDA adolescents, the prevalence of family level ACEs such as parental substance misuse (26.3%) and domestic violence (37.2%) was notably high, and these adolescents were four times more likely to misuse TAC after adjustments for confounders. Adolescents may be more vulnerable to family level ACEs living within a cultural context where family relationships are valued more than individual autonomy and where parental substance misuse is considered a family disease, strongly associated with societal stigma [38, 39]. This is supported by the results of our IPD meta-analysis, where we found clear ACE-TAC association at child level and collective level. For example, in NIMHANS, where children of parents attending de-addiction clinics were recruited and with experience of at least one family level ACE, adolescents were almost seven times more likely to misuse TAC. The overlap between substances misused by adolescents and those misused by parents is limited and is a caveat to the data collected. Nevertheless, the intergenerational effects of substance misuse have been documented with substance misuse impacting on parenting and the effects of dysfunctional parenting on offspring [40]. Political conflict may drive other kinds of childhood adversities and have tangible negative effects on children and their development leading to anxiety, depression, poor school performance and experimentation with substances [16]. In Imphal, experiencing such collective level ACEs are associated with a five-fold increased risk of TAC misuse, and TAC is more prevalent in males (15.9%) than in females (4.2%) in the city. Tobacco is the primary substance used most harmfully and hazardously (Additional file 5: Appendix 5) and our figures correspond with published estimates that 5–25% of Indian adolescents currently use or have used tobacco in various forms (e.g. chewing and smoking) [16].

In cVEDA young adults, the prevalence of child level ACEs (48%) such as abuse and family level ACEs (47%) such as domestic violence was notably high whilst TAC was more prevalent in males (18.3%) than females (2%). After adjustment for confounders, cVEDA young adults with experience of child maltreatment ACEs were twice as likely to misuse TAC (adjusted OR 1.96; 95% CI 1.10–3.49), whilst the results were weaker for family level ACEs (unadjusted OR 4.05, 95% CI 2.97–5.52, adjusted OR 1.58, 95% CI 0.89–2.78). Child maltreatment levels were comparable in adult males and females (48% vs. 47%) but are notably higher compared to other cohort studies globally: 29% in the Netherlands [41]; 0.5% in the United Kingdom [42]. The majority of child maltreatment literature is concentrated in North America or Europe and often, results are extrapolated, inaccurately, to the world population. A consistent theme in child maltreatment studies across continents is the high prevalence of emotional abuse compared with physical and sexual abuse and this is reflected in cVEDA adolescents (emotional abuse: 44.2%; physical abuse: 36.4%; sexual abuse: 1.5%) and young adults (emotional abuse: 39.8%; physical abuse: 28.4%; sexual abuse: 3.1%).

Such ACEs are key risk factors in maladaptive coping strategies which increase vulnerability to and adoption of negative health behaviours such as alcohol, tobacco and illicit drug consumption [3, 4, 6]. Similar to cVEDA, previous authors have shown underage consumption of alcoholic drinks affecting males more than females. Males were subsequently more likely than females to misuse TAC (adolescents: 5% of males, 0.8% of females; young adults: 18.3% of males; 2% of females). Tobacco use in males is higher than females and often occurs concurrently with alcohol use [38, 42], trends reflected in cVEDA. The disproportionate ACE occurrence by sex may explain the disproportionate sex ratio in substance misuse particularly for tobacco and alcohol [43]. More TAC in males can also be attributed to the reduced social acceptability of smoking in females; reduced likelihood of admitting use of substances and preference for alternative substances such as smokeless tobacco products [44].

### Strengths and Limitations

This is the first national study undertaken in India investigating the effects of ACEs on substance misuse in a paediatric population. Furthermore, the case definitions used for ACEs and substance misuses (both using WHO-endorsed assessment tools), provides further ratification to our study methodology. Also, our conservative approach in using a random-effects meta-analysis, whilst producing wider confidence intervals than a fixed-effects method, allows us to reduce any type 1 error rates, a distinct advantage in our study design [30]. However, there are several caveats to our work. The cross-sectional nature of the study design is a limitation as we cannot determine the nature and direction of association between ACEs and harmful and hazardous use of TAC. As cVEDA is a longitudinal cohort study with follow up assessments [13, 20], we may be able to better determine the strength and association of such relationships in the coming years. Secondly, the recall of ACEs using the ACEIQ questionnaire, a WHO questionnaire designed for use in adults aged 18 years and over [22] is a caveat. ACEIQ use in cVEDA adolescents may be limited as even if individuals are willing and able to recount childhood adversities, they may not always be aware of their occurrence [37]. Furthermore, prospective recall of ACEs are considered to be more valid but have lower sensitivity (identify small prevalence of maltreated individuals) whilst retrospective measures show strong associations with self-reported outcomes rather than objectively ascertained outcomes, indicating common method bias [45]. Thirdly, we used the traditional ACEs categories often used in the evidence base [2, 3, 5] but did not include extended ACEs such as financial difficulties, or social support of the parent as these data were not collected. In LMICs particularly, ACE constructs should be uniquely considered to include poverty, as it is a result of family economic circumstance and is often associated with ACEs [46]. Lastly, whilst the study focussed on commonly used substances (alcohol, tobacco and cannabis), these were all self-reported in the cohort. Underreporting of use of illicit substances is possible given the nature of the study design (e.g. children and adolescents may have been interviewed with parents/guardians in the vicinity) and stigma and taboo associated with using and admitting to illegal substances [39].

### Policy Implications

Robert Anda described the circumstance created by ACEs as a ‘chronic public health disaster’ [3]. As ACEs are highly prevalent and strongly related to substance misuse outcomes, preventing ACEs and identifying children who have had such experiences, could help identify where effort and intervention may be most needed. Specific considerations should include, collating more state- and country level ACEs data to help inform local decision making (e.g. by incorporating ACE enquiries into nationwide cohort studies and subsequent follow-up); including ACEs among risk factors and protective factors when planning prevention based interventions, and, using ACEs research and local ACEs expertise to identify groups of children or young people who may be at higher risk for substance misuse so that targeted prevention plans can be created [47].

For example, enabling ACE prevention in India requires selecting effective policies, for e.g. encouraging positive disciplining instead of corporal punishment. This would help distinguish behaviour that is punishment focussed rather than improvement or learning focussed for a child. This approach has had success as part of the ‘Safe Families’ scheme in the Philippines [48] which seeded changes to the cultural norm of physical punishment. Other authors [49] recommend that child maltreatment interventions should focus on the whole family to reduce the intergenerational transmission of family violence. Given the intergenerational transmission of substance misuse too, this type of intervention would address both the exposure and outcome of interest from our study design.

Given that tobacco is the substance most used harmfully and hazardously in cVEDA and tobacco addiction in adults is often initiated during childhood, it is imperative that tobacco control measures targeting young people, are improved [19]. Policies regulating smoking in public spaces and tobacco selling should be implemented effectively and should include awareness programmes for harmful effects [50]. These awareness programmes should begin at grassroots levels in local communities, building societies or schools so that the message is conveyed at an early age to children and adolescents.

Further research in the area requires a focus on standardising criteria for evaluating ACEs at a population level and, examination of the heterogeneity of substance types included in substance misuse research. Further work in this area will elucidate the mechanism of the relationship between ACEs and substance misuse [51, 52].

## Conclusions

Child maltreatment and domestic violence are the most common forms of ACEs in cVEDA. Harmful and hazardous use of tobacco was the most common type of substance misuse in adolescents and young adults. Given early childhood adversities shape cognitive schemas (defence mechanisms) and subsequent behaviours, health professionals, particularly in addiction services, need to be sensitised and trained (trauma-informed) to deal with the long-term impact of ACEs on cognitive, emotional and behavioural functioning.

## Supplementary Information

**Additional file 1: Appendix 1**. Breakdown of hazardous use of substances by age band in cVEDA.

**Additional file 2: Appendix 2**. ACEs by recruitment centres in cVEDA.

**Additional file 3: Appendix 3**. Breakdown of hazardous use of tobacco, alcohol and cannabis by cVEDA site.

**Additional file 4: Appendix 4**. Unadjusted and adjusted effect sizes and heterogeneity measures for ACE levels and tobacco, alcohol, cannabis outcomes in adolescents and young adults.

**Additional file 5: Appendix 5**. 102 adolescents had one form of substance misuse at least. We used *pvenn* in STATA to present the overlap of substances.

## Data Availability

The data that support the findings of this study are available but restrictions apply to the availability of these data, which were used under license for the current study, and so are not publicly available. Data are however available from the authors upon reasonable request, submission of a data access form and with permission from the study senior team and PI’s, Professor Gunther Schumann and Professor Vivek Benegal.

## Abbreviations

ACE: Adverse childhood experiences
ACEIQ: Adverse childhood experience international questionnaire
WHO: World Health Organisation
TAC: Tobacco, Alcohol and Cannabis
IPD: Individual Patient Data
cVEDA: Consortium on Vulnerability to Externalising Disorders and Addictions
ASSIST: Alcohol, Smoking and Substance Involvement Screening Test
NIMHANS: National Institute of Mental Health and Neurosciences
PGIMER: Post Graduate Institute of Medical Education and Research
SES: Socioeconomic Status
HKSJ: Hartung-Knapp-Sidik-Johnkman
OR: Odds Ratio
CI: Confidence Intervals
PMLE: Penalised maximum likelihood regression
