# Supplementary material for: Adverse childhood experiences and substance misuse in young people in India: results from the multisite cVEDA cohort
Source: BMC Public Health. 2021 Oct 23;21:1920. doi: 10.1186/s12889-021-11892-5 (PMC8539836; doi:10.1186/s12889-021-11892-5)
Supplement: Supplementary file 1 — Additional file 1: Appendix 1. Breakdown of hazardous use of substances by age band in cVEDA. [file 12889_2021_11892_MOESM1_ESM.docx]

Appendix 1. Breakdown of hazardous use of substances by age band in cVEDA

|  | TAC  (n, %) | Tobacco  (n, %) | Alcohol  (n, %) | Cannabis  (n, %) |
| --- | --- | --- | --- | --- |
| Children (C1) | 2 (0.07) | 2 (0.08) | 0 | 0 |
| Adolescents (C2) | 102 (3.03) | 92 (2.90) | 26 (0.82) | 47 (1.48) |
| Young Adults (C3) | 242 (8.80) | 209 (8.07) | 107 (4.13) | 66 (2.55) |
