# Supplementary material for: Adverse childhood experiences and substance misuse in young people in India: results from the multisite cVEDA cohort
Source: BMC Public Health. 2021 Oct 23;21:1920. doi: 10.1186/s12889-021-11892-5 (PMC8539836; doi:10.1186/s12889-021-11892-5)
Supplement: Supplementary file 2 — Additional file 2: Appendix 2. ACEs by recruitment centres in cVEDA. [file 12889_2021_11892_MOESM2_ESM.docx]

Appendix 2. ACEs by recruitment centres in cVEDA

| **Type of ACE** | **Imphal**  n (%) | **Asansol**  n (%) | **Mysore**  n (%) | **NIMHANS**  n (%) | **PGIMER**  n (%) | **Rishi Valley**  n (%) | **St. John’s Research Institute** n (%) |
| --- | --- | --- | --- | --- | --- | --- | --- |
| Physical Abuse | 951 (85.75) | 79 (6.52) | 189 (14.30) | 884 (48.68) | 224 (21.79) | 21 (2.72) | 524 (55.39) |
| Sexual Abuse | 3 (2.38) | 25 (2.06) | 7 (1.53) | 79 (4.36) | 44 (4.29) | 3 (0.39) | - |
| Emotional Abuse | 921 (83.05) | 142 (11.72) | 350 (26.44) | 1184 (65.16) | 245 (23.83) | 69 (8.94) | 678 (71.67) |
| Emotional Neglect | 234 (21.10) | 107 (8.81) | 138 (9.96) | 240 (13.19) | 124 (12.07) | 33 (4.27) | 152 (16.10) |
| Physical Neglect | 282 (25.43) | 45 (3.71) | 58 (4.20) | 229 (12.62) | 132 (12.87) | 36 (4.66) | 21 (2.22) |
| ***Child Level**** | 1065 (94.92) | 240 (15.73) | 457 (32.34) | 1314 (69.67) | 372 (29.34) | 131 (16.88) | 760 (74.58) |
| Parental Divorce | 223 (19.88) | 42 (2.75) | 56 (4.01) | 174 (9.23) | 62 (4.96) | 76 (9.79) | 70 (6.88) |
| Parental Mental Illness | 99 (8.94) | 10 (0.82) | 15 (1.09) | 131 (7.21) | 133 (12.94) | 5 (0.65) | 9 (0.95) |
| Parental Substance Abuse | 276 (24.89) | 271 (22.29) | 139 (10.17) | 918 (50.50) | 614 (59.79) | 82 (10.62) | 50 (5.29) |
| Parental Incarceration | 55 (4.96) | 11 (0.90) | 5 (0.37) | 29 (1.60) | 19 (1.85) | 12 (1.55) | 11 (1.16) |
| Domestic Violence | 928 (82.86) | 111 (7.29) | 199 (14.28) | 892 (47.35) | 338 (27.06) | 128 (16.49) | 379 (37.30) |
| ***Family Level**** | 961 (85.65) | 355 (23.26) | 326 (23.07) | 1199 (63.57) | 762 (60.09) | 203 (26.16) | 432 (42.49) |
| Bullying | 178 (16.08) | 22 (1.81) | 194 (14.15) | 203 (11.19) | 116 (11.34) | 23 (2.98) | 27 (2.85) |
| Community Violence | 881 (78.52) | 54 (3.54) | 242 (17.30) | 352 (18.68) | 209 (16.72) | 275 (35.44) | 265 (26.08) |
| ***Community Level**** | 921 (82.09) | 72 (4.72) | 309 (21.87) | 471 (24.97) | 274 (21.61) | 290 (37.37) | 280 (27.48) |
| Collective Violence | 226 (20.16) | 3 (0.20) | 29 (2.09) | 39 (2.07) | 21 (1.68) | 15 (1.93) | 5 (0.49) |
