# Supplementary material for: Adverse childhood experiences and substance misuse in young people in India: results from the multisite cVEDA cohort
Source: BMC Public Health. 2021 Oct 23;21:1920. doi: 10.1186/s12889-021-11892-5 (PMC8539836; doi:10.1186/s12889-021-11892-5)
Supplement: Supplementary file 3 — Additional file 3: Appendix 3. Breakdown of hazardous use of tobacco, alcohol and cannabis by cVEDA site. [file 12889_2021_11892_MOESM3_ESM.docx]

Appendix 3. Breakdown of hazardous use of tobacco, alcohol and cannabis by cVEDA site

|  | **Imphal**  n (%) | **Asansol**  n (%) | **Mysore**  n (%) | **NIMHANS**  n (%) | **PGIMER**  n (%) | **Rishi Valley**  n (%) | **St. John’s Research Institute** n (%) |
| --- | --- | --- | --- | --- | --- | --- | --- |
| Tobacco  (n, %) | 99 (8.89) | 32 (.247) | 22 (1.57) | 125 (6.83) | 24 (2.31) | 1 (0.13) | 0 |
| Alcohol  (n, %) | 38 (3.41) | 8 (0.62) | 13 (0.93) | 57 (3.12) | 15 (1.44) | 2 (0.26) | 0 |
| Cannabis  (n, %) | 36 (3.23) | 8 (0.62) | 0 | 53 (2.90) | 16 (1.54) | 0 | 0 |
