# Supplementary material for: Adverse childhood experiences and substance misuse in young people in India: results from the multisite cVEDA cohort
Source: BMC Public Health. 2021 Oct 23;21:1920. doi: 10.1186/s12889-021-11892-5 (PMC8539836; doi:10.1186/s12889-021-11892-5)
Supplement: Supplementary file 4 — Additional file 4: Appendix 4. Unadjusted and adjusted effect sizes and heterogeneity measures for ACE levels and tobacco, alcohol, cannabis outcomes in adolescents and young adults. [file 12889_2021_11892_MOESM4_ESM.docx]

Appendix 4. Unadjusted and adjusted effect sizes and heterogeneity measures for ACE levels and tobacco, alcohol, cannabis outcomes in adolescents and young adults

| ACE level | Unadjusted Overall Effect (OR) | Unadjusted  95% CI | Adjusted Overall Effect (OR) | Adjusted  95% CI | Cochrane’s Q  *Q df p value* | | | I^2^  ^(%)^ | tau^2^ |
| --- | --- | --- | --- | --- | --- | --- | --- | --- | --- |
| *Adolescents*  ***Tobacco***  Child level | 3.29 | 1.83-5.94 | 3.49 | 0.13-94.82 | 5.82 | 2 | 0.08 | 61.4 | 0.99 |
| Family level | 3.68 | 1.95-6.92 | 3.92 | 1.38-11.12 | 2.66 | 3 | 0.44 | 0 | 0.00 |
| Community level | 3.11 | 1.95-4.98 | 2.85 | 0.76-10.67 | 0.47 | 2 | 0.79 | 0 | 0 |
| Collective level | 6.97 | 4.15-11.70 | 8.43 | 0.95-74.40 | 3.75 | 2 | 0.15 | 46.7 | 0.36 |
| ***Alcohol***** |  |  |  |  |  |  |  |  |  |
| Child level | Insufficient data | - | - | - | - | - | - | - | - |
| Family level | - | - | - | - | - | - | - | - | - |
| Community level | - | - | - | - | - | - | - | - | - |
| Collective level | - | - | - | - | - | - | - | - | - |
| ***Cannabis*** |  |  |  |  |  |  |  |  |  |
| Child level | 5.20 | 2.27-11.95 | 2.88 | 0.38-22.08 | 5.82 | 3 | 0.12 | 48.4 | 0.79 |
| Family level | 4.19 | 1.53-11.42 | 3.91 | 0.00-3651.9 | 0.58 | 1 | 0.45 | 0 | 0 |
| Community level | 2.63 | 1.43-4.83 | 2.69 | 0.01-524.4 | 0.27 | 1 | 0.61 | 0 | 0 |
| Collective level | 8.58 | 4.53-16.25 | 9.74 | 1.67-56.87 | 0.48 | 2 | 0.79 | 0 | 0 |
| *Young Adults*  ***Tobacco***  Child level | 4.42 | 3.10-6.29 | 1.93 | 1.03-3.67 | 2.89 | 4 | 0.58 | 0 | 0.00 |
| Family level | 3.32 | 2.40-4.60 | 1.26 | 0.69-2.31 | 2.36 | 4 | 0.67 | 0 | 0.00 |
| Community level | 2.63 | 1.97-3.50 | 1.46 | 0.70-3.27 | 7.84 | 4 | 0.09 | 49 | 0.20 |
| Collective level | 3.38 | 2.25-5.10 | 2.67 | 0.08-86.16 | 0 | 1 | 0.95 | 0 | 0 |
| ***Alcohol*** |  |  |  |  |  |  |  |  |  |
| Child level | 4.27 | 2.51-7.27 | 2.52 | 0.94-6.78 | 1.6 | 3 | 0.66 | 0 | 0 |
| Family level | 4.73 | 2.75-8.14 | 2.56 | 0.57-11.46 | 4.95 | 3 | 0.18 | 39.4 | 0.31 |
| Community level | 2.40 | 1.63-3.56 | 1.49 | 0.37-5.95 | 11.94 | 4 | 0.02 | 66.5 | 0.60 |
| Collective level | 3.65 | 2.18-6.10 | 2.62 | 0.03-210.9 | 0 | 1 | 0.95 | 0 | 0 |
| ***Cannabis*** |  |  |  |  |  |  |  |  |  |
| Child level | 2.44 | 1.28-4.65 | 1.47 | 0.40-5.38 | 3.19 | 3 | 0.36 | 5.9 | 0.04 |
| Family level | 1.93 | 0.98-3.77 | 1.42 | 0.40-4.92 | 3.22 | 3 | 0.36 | 6.7 | 0.04 |
| Community level | 1.16 | 0.71-1.90 | 1.22 | 0.40-3.66 | 3.43 | 3 | 0.33 | 12.6 | 0.07 |
| Collective level | 2.59 | 1.39-4.82 | 2.37 | - | 2.92 | 1 | 0.09 | 65.8 | 0.66 |

**Alcohol misuse frequency is 26 in this age band
