# Supplementary material for: Adverse childhood experiences and substance misuse in young people in India: results from the multisite cVEDA cohort
Source: BMC Public Health. 2021 Oct 23;21:1920. doi: 10.1186/s12889-021-11892-5 (PMC8539836; doi:10.1186/s12889-021-11892-5)
Supplement: Supplementary file 5 — Additional file 5: Appendix 5. 102 adolescents had one form of substance misuse at least. We used pvenn in STATA to present the overlap of substances. [file 12889_2021_11892_MOESM5_ESM.docx]

Appendix 5.

102 adolescents had one form of substance misuse at least. We used *pvenn* in STATA to present the overlap of substances:

*
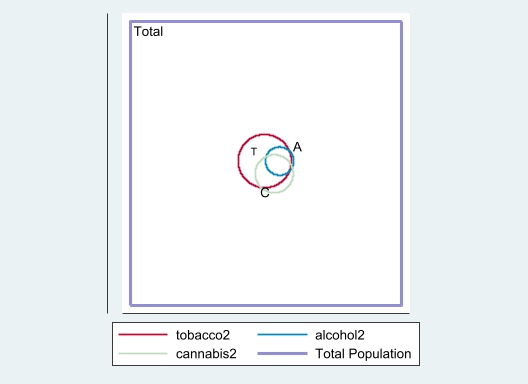
*

*(A) tobacco (T): 92*

*(B) alcohol (A): 26*

*(C) cannabis (C): 47*

*AB Overlap: 26*

*AC Overlap: 37*

*BC Overlap: 17*

*ABC Overlap:17*

For the young adult group, 242 had at least one form of substance misuse.

1. *
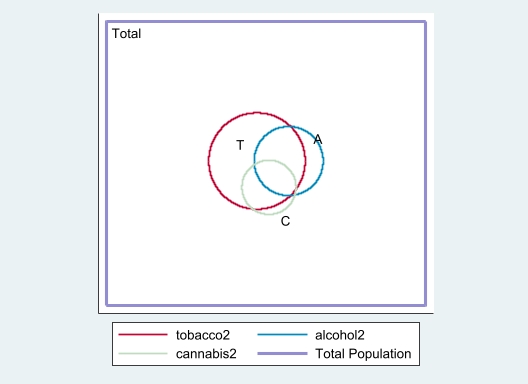
Tobacco (T): 209*
2. *Alcohol (A): 107*
3. *Cannabis (C): 66*

*AB Overlap: 78*

*AC Overlap: 60*

*BC Overlap: 30*

*ABC Overlap: 28*
